# Diabetes educational interventions in care homes: a scoping review

**DOI:** 10.1186/s12909-026-08738-2

**Published:** 2026-02-05

**Authors:** Kathie-Anne Walker, Stephanie Craig, Tara Anderson, Patrick Stark, Christine Brown Wilson, Gillian Carter, Claire McEvoy, Laura Creighton, Elizabeth Henderson, Shannon Porter, Fadwa Alhalaiqa, Erin Ferranti, Komal Patel Murali, Yaguang Zheng, Roberta Sammut, Marwa Mamdouh Shaban, Hon Lon Tam, Norbert Buzás, Don Leidl, Gary Mitchell

**Affiliations:** 1 School of Nursing and Midwifery, Queen’s University Belfast, Belfast, Northern Ireland; 2 Queen’s University Belfast, Centre for Public Health, Belfast, Northern Ireland; 3 College of Nursing, Qatar University, Doha, Qatar; 4 Emory University, Nell Hodgson Woodruff School of Nursing, Atlanta, GA USA; 5 College of Nursing, New York University, New York, United States of America; 6 Faculty of Health Sciences, University of Malta, Msida, Malta; 7 Faculty of Nursing, Cairo University, Cairo, Egypt; 8 The Nethersole School of Nursing, The Chinese University of Hong Kong, Hong Kong , SAR China; 9 Faculty of Health Sciences and Social Studies, University of Szeged, Szeged, Hungary; 10 Faculty of Nursing, University of New Brunswick, Moncton, Canada

**Keywords:** Diabetes, Older people, Scoping review, Care homes, Education, Educational interventions, Aged care facilities, Nursing care

## Abstract

**Background:**

Diabetes affects approximately 10.5% of the global adult population and is more prevalent in care homes due to residents’ advanced age and multimorbidity. Effective diabetes management in these settings is essential to prevent complications and maintain quality of life, yet evidence addressing the specific needs of this population remains limited. High-quality care relies on access to appropriate clinical education. This scoping review will synthesise evidence on educational interventions to support diabetes care provision in care home settings.

**Methods:**

This scoping review was undertaken in accordance with the Preferred Reporting Items for Systematic Reviews and Meta-Analyses extension for Scoping Reviews (PRISMA-ScR) guidelines. A comprehensive literature search was conducted across three electronic databases: CINAHL Plus, Medline, and PsycINFO. Methodological quality of the included primary studies was assessed using the Mixed Methods Appraisal Tool (MMAT).

**Results:**

In total, 10 studies were included in the review, encompassing evidence from a range of international contexts. Analysis revealed three prominent themes; Firstly, educating nurses about diabetes can improve practice and behaviour. Secondly, educational interventions can increase staff knowledge and confidence, which is linked to enhancing the quality of care. Finally, a range of facilitators and barriers influencing the delivery of diabetes training in care homes were identified.

**Discussion:**

The review suggests that educational interventions in care homes can enhance diabetes care. However, while the current evidence is encouraging, there are a lack of empirically tested educational interventions for diabetes education in this setting. Further, current educational programmes appear to lack key detail including footcare, eye care and COVID-19. To ensure the provision of high-quality diabetic care, it is therefore important to enhance the training and education of staff members.

## Introduction

Diabetes is a significant global health concern, with approximately 537 million people affected worldwide [1]. Of these, the majority live in low- and middle-income countries, where the prevalence is rapidly increasing due to factors such as ageing populations and lifestyle changes [1]. In the United Kingdom, 4.3 million individuals are currently diagnosed with diabetes, with this number projected to rise to 5.5 million by 2030[2]. Despite advances in medical care and public health education, diabetes remains a persistent challenge, especially as life expectancy continues to rise globally [1]. The condition is expected to affect double the current number of people by 2040 and more than triple by 2050[3].

While recent research indicates a decline in diabetes prevalence in some age groups due to public health interventions [4], the overall trend remains concerning. Diabetes is a chronic condition characterised by elevated blood glucose levels resulting from insufficient insulin production or use. The disease presents a range of complications, including cardiovascular disease, neuropathy, retinopathy, and nephropathy [1]. These complications often worsen with age and require careful management to avoid severe outcomes such as cardiovascular death, which accounts for approximately 20% of diabetes-related mortality globally [1]. Given the growing number of individuals affected, and the complex management needs of older adults with diabetes, particularly those in care homes, addressing this issue has become an urgent public health priority.

The economic burden of diabetes is significant, with global healthcare costs related to the condition estimated to reach USD 2.5 trillion by 2030[5]. In the UK, diabetes costs the National Health Service (NHS) an estimated £14 billion annually, highlighting the financial strain of managing this chronic condition [6]. Care home residents, who are often older and have multiple comorbidities, represent a significant portion of this expenditure. As the number of individuals with diabetes in care homes continues to grow, it is imperative that healthcare systems invest in effective diabetes management strategies that reduce complications and improve health outcomes.

Given the growing prevalence of diabetes in care homes [7], there is an increasing need for targeted educational interventions aimed at care home staff. These interventions are essential in equipping staff with the knowledge and skills to manage diabetes effectively in a population that is particularly vulnerable to the complications of the disease. According to the National Advisory Panel on Care Home Diabetes [7], comprehensive staff training in diabetes management, individualised care plans, and regular health assessments are crucial components of effective diabetes care in these settings. However, despite the importance of staff education, it remains unclear to what extent educational interventions have been developed and tested for diabetes management in care homes.

Therefore, the aim of this review is to explore the existing educational interventions for diabetes management in care homes, assess their effectiveness, and identify gaps in current research.

### Aim and objectives

The aim of this review was to synthesise evidence on educational interventions related to diabetes care in care homes. The specific objectives were:

1. Summarise the existing educational and training interventions available to staff in care homes regarding diabetes management.
2. Explore the outcomes of educational interventions employed in training staff within care home environments on diabetes care.
3. Identify gaps and limitations in current educational interventions for staff in care homes concerning diabetes care.

## Methods

### Study design

This study adopted a scoping review methodology to systematically examine the existing literature on educational interventions for diabetes care in care home settings. The review was conducted in line with methodological guidance from the Joanna Briggs Institute [8], and reported in accordance with the Preferred Reporting Items for Systematic Reviews and Meta-Analysis extension for ScR (PRISMA-ScR) [9]. The review process comprised the development of a clearly defined review focus, the establishment of eligibility criteria, and the identification and selection of relevant studies through structured searching and screening. Data were charted and synthesised to provide a descriptive and narrative overview of the available evidence. A protocol for this scoping review was not registered, as protocol registration is not mandatory for scoping reviews.

### Search strategy

The search strategy was developed collaboratively by the review team, with specialist input from an academic librarian. To inform the development of search terms and gain an overview of the existing literature, an initial exploratory search was conducted using Google Scholar, focusing on diabetes education within care home settings.

Searches were conducted in three electronic databases: CINAHL Plus (EBSCOhost), MEDLINE All (Ovid), and PsycINFO (Ovid). These databases were selected because they index literature across nursing, medicine, allied health, psychology, and the social sciences. The databases were searched in December 2024. Search strategies were adapted for each database using a combination of controlled vocabulary (e.g., MeSH terms where applicable) and free-text keywords. Core concepts included “diabetes”, “care home/nursing home/long-term care”, and “education/training”, with database-specific modifications made in line with indexing conventions; an example strategy is provided in Table 1. In addition, reference lists of included studies were checked to identify any further eligible papers, as recommended by Horsley et al.[10] All records were imported into Covidence for screening and study management (https://www.covidence.org).

**Table 1:** Example of search terms

Key words (*P*)		Key words (E)		Key words (O)
Diabetes*	AND	Residential Home*	AND	Education
OR		OR		OR
Type 2 Diabetes		Nursing Home*		Educational activities
OR		OR		OR
Type 1 Diabetes		Long term care facility*		Nursing Education
OR		OR		OR
Diabetes Mellitus		Care Home*		Training
OR		OR		OR
Diabetic		Aged Care facility*		Self-directed learning
		OR		OR
		Care Home staff		E-Learning
		OR		OR
		Nursing home staff		Staff Training
		OR		CPD
		Residential home staff		OR
				Continued professional development
				OR
				Educational intervention

### Inclusion/exclusion criteria

Inclusion criteria were developed using the Population, Exposure and Outcome (PEO) framework, as outlined by Bettany-Saltikov and McSherry [11]. The review considered a wide range of empirical study designs, including randomised controlled trials, quasi-experimental studies, cohort studies, case–control studies, cross-sectional studies, qualitative research, and mixed-methods studies. Only articles published in English were included; no geographical or publication date limits were applied.

The population of interest comprised staff working in care home settings who were involved in the provision of direct resident care. Eligible studies were required to be conducted within care home contexts, including nursing homes, residential care facilities, assisted living facilities, and other long-term care environments. Skilled nursing facilities and short-term rehabilitation settings were excluded. Where educational or training interventions addressed diabetes alongside other conditions, only findings explicitly related to diabetes care were extracted and analysed. Studies undertaken in hospital or community settings, or those focusing on healthcare professionals outside the care home workforce, were excluded. A detailed summary of the inclusion and exclusion criteria is presented in Table 2.

**Table 2:** Inclusion and exclusion criteria

Inclusion	Exclusion
Empirical research on diabetes education in care homes	Non-English articles
Literature reviews, theses, dissertations or conference abstracts	Commentary papers
Studies that include an educational intervention	People other than staff employed in care homes such as doctors, physiotherapists, pharmacists, dieticians or dentists.
Articles written in English	Studies that do not include an educational intervention
No time restrictions	

### Study selection

All records retrieved from the database searches were uploaded into Covidence for management and screening. Titles and abstracts were independently reviewed by two reviewers (KW & GM), followed by full-text screening of studies meeting the initial eligibility criteria. Any disagreements or conflicts arising during either stage of screening were resolved through discussion, with input from a third reviewer (SC) where consensus could not be reached.

### Data extraction

Data extraction focused on key study characteristics, including participant groups, care home context, features of the educational interventions, outcome domains assessed, and principal findings. Extraction was conducted directly within Covidence. Data were charted descriptively, with no assumptions, simplifications, or transformations applied, as findings were synthesised narratively rather than through statistical aggregation. Extracted information included details relating to participants, concepts, contextual factors, study design, and outcomes relevant to the review question.

Of the six quantitative studies included, five employed non-randomised experimental designs and one was a randomised controlled trial. Although critical appraisal is not a mandatory component of scoping reviews, it is recommended within Item 12 of the PRISMA-ScR Checklist in item 12 (https://www.prisma-statement.org/scoping). Incorporating a quality appraisal enables readers to better contextualise and interpret the strength and reliability of the included evidence, recognising that conclusions drawn from higher-quality studies may carry greater confidence than those derived from lower-quality research⁹. All studies were retained in the review regardless of methodological quality, allowing for a transparent overview of the quality of the available evidence base.

### Data analysis

Data were synthesised using a narrative approach, which is well suited to the integration of evidence derived from studies with diverse designs and outcome measures [12]. Following data extraction, studies were systematically compared to identify patterns, contrasts, and relationships across findings, with attention given to the scope, consistency, and strength of the reported evidence. To facilitate this process, data aligned with the review objectives, such as characteristics of the educational interventions, modes of delivery, and reported outcomes, were charted and organised using an initial analytical framework. These data were then iteratively grouped into themes to support a structured and coherent synthesis of the findings.

Findings were reported within these themes using narrative and descriptive methods [13, 14]. The synthesis was undertaken collaboratively by three reviewers (KW, SC, and GM), with extracted data and emerging themes reviewed iteratively. Any differences in interpretation were resolved through discussion until consensus was reached. Consistent with guidance from Levac et al. [15], the final themes were interpreted in relation to the review objectives, namely to map existing educational and training interventions, examine their reported outcomes, and identify gaps and limitations in diabetes education for staff working in care home settings.

## Results

**Fig. 1:**
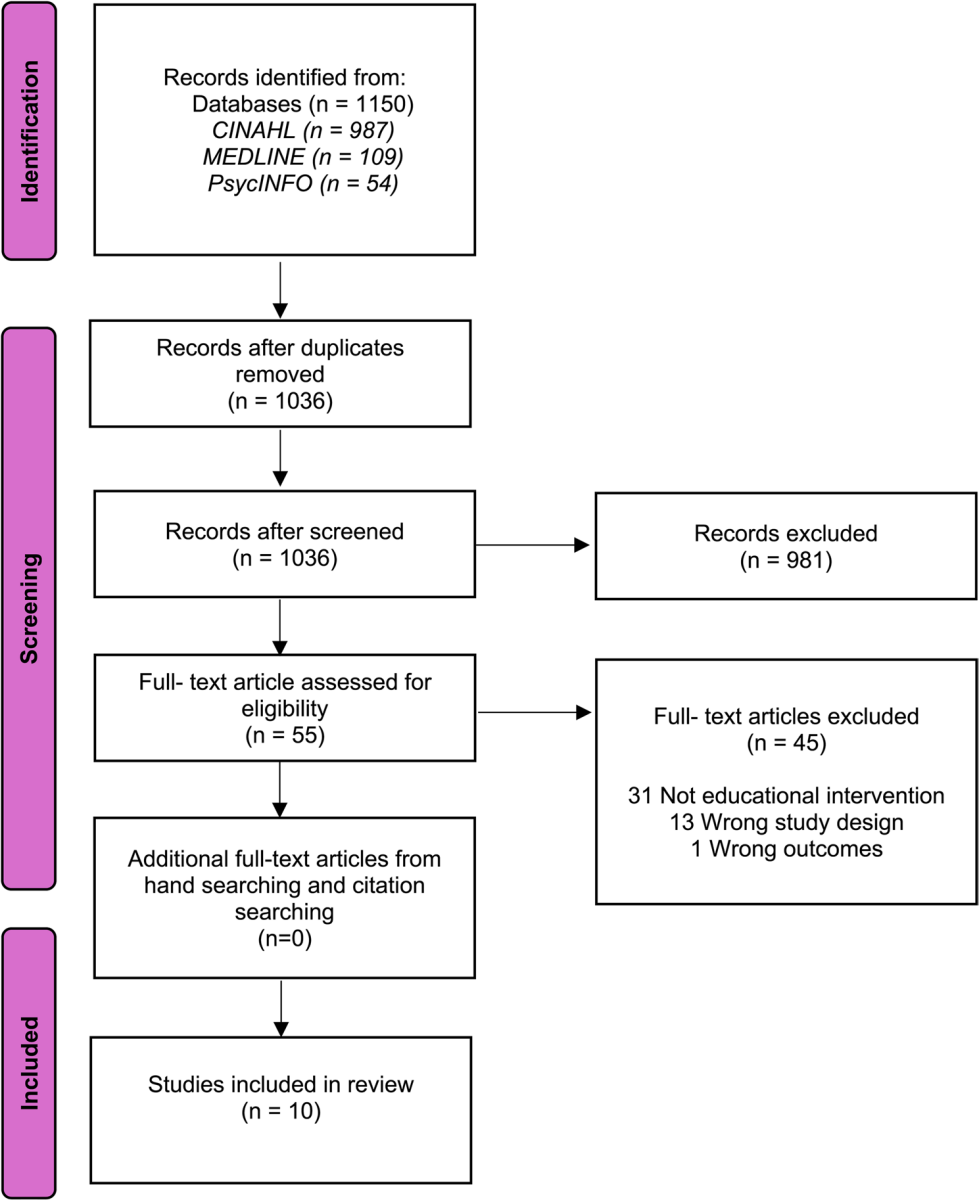
PRISMA- ScR flow chart summarising the search process

After the database searches, a total of 1,150 were found. Figure 1 shows a PRISMA- ScR flow chart summarising the search process. Following abstract and title review, 55 papers remained for full text review. In total 45 papers were excluded. Resulting in, 10 articles being included in the final review (Deakin et al.[16], Gregory [17], Hare [18], Lega et al.[19], and Munshi et al. [20], Parker et al.[21], Hausken et al.[22], Heeley-Creed et al. [23], Scott et al. [24], and Whylie-Rosett et al.[25]. These studies were identified as eligible based on the inclusion criteria and on the primary aim for the review.

### Study characteristics

Six studies in this review used a quantitative approach, five were experimental studies without randomisation [16–20] and one was a randomised controlled study [21]. Additionally, four studies [22–25] utilised a mixed methods approach. Details of study settings can be seen in Table 2. Four studies were conducted in England [16–18, 23], three in the United States (USA) [20]– [21, 25], one in Scotland [24], one in Canada [19] and one in Norway [22].

**Table 3:** Study details

Authors/Country	Intervention	MMAT Score	Aim of research	Research design	Sample and setting	Main findings
Deakin et al., 2001[16] (England)	Face-to-face diabetes education delivered to care home staff, consisting of classroom teaching supported by practical demonstrations relevant to daily care.	5	To deliver an educational programme to care home staff in one residential home and assess its impact on staff knowledge and practice.	Quantitative Non RCT	1 Residential home	• Significant pre–post improvement in staff diabetes knowledge • Knowledge retention of 92% at 12 months • Improvements reported in diabetes care practices, including glucose monitoring and care routines • No resident-level clinical outcomes measured
Gregory, 2018 [17] (England)	Face-to-face diabetes training aligned to Diabetes UK standards, focusing on insulin administration, supported by collaboration with community nursing teams and telecare in some homes.	3	To encourage staff to meet basic diabetes standards set out by Diabetes UK (2010) report.	Quantitative Non RCT	33 care homes	• Increased staff compliance with Diabetes UK minimum standards • 90% of unregistered practitioners delegated insulin administration post-training • Reduction in community nursing visits by 378 per week • Reduction in hospital admissions (65%) and emergency calls (69%) related to diabetes
Hare,1997[18] (England)	Face-to-face diabetes education sessions for nursing home staff covering core aspects of diabetes care, delivered as structured teaching sessions.	4	To improve the care of people with diabetes in Bath nursing homes.	Quantitative Non RCT	9 Nursing homes	• Increase in staff diabetes knowledge ranging from 7% to 30% • Knowledge improvements in diet, insulin use, hypoglycaemia, illness management, and foot care • Decrease in knowledge related to diabetes monitoring • No behavioural or resident clinical outcomes assessed
Hausken et al., 2013[22] (Norway)	Face-to-face diabetes training programme combining formal teaching with facilitated peer discussion and experience exchange for nurses and nursing aides.	4	To outline the creation and execution of an educational training programme focused on diabetes care and management for registered nurses and nursing aides working in nursing homes and municipal home-based services. Additionally, this report aims to present the evaluation of the programme and the feedback from the participants.	Mixed Methods	1 Nursing home	• Improved staff knowledge, confidence, and professional development following training • Clinically significant increases (5–10 points) in confidence related to diabetes care • Enhanced ability to advocate for patients and apply evidence-based care • Limited time identified as the main barrier to engagement
Heeley-Creed et al., 2007[23] (England)	On-site, face-to-face diabetes training delivered across nursing and residential care homes, supported by printed preparatory materials to encourage attendance.	3	To raise awareness of optimal diabetes care among staff in nursing and residential care homes and aims to provide a comprehensive training program for all staff working with elderly residents.	Mixed Methods	14 Nursing homes 75 Residential homes	• Increased staff awareness of optimal diabetes care across participating homes • High staff engagement, with 936 staff attending training sessions • Evidence-based, tailored training associated with perceived improvements in diabetes care quality • No resident-level clinical outcomes reported
Lega et al., 2020[19] (Canada)	Structured face-to-face education sessions for frontline long-term care staff focused on practical diabetes management topics.	5	To assess the effectiveness of an educational intervention on the comfort and knowledge of diabetes management among frontline long-term care staff.	Quantitative Non RCT	2 LTC facilities	• Significant improvements in staff knowledge and comfort managing diabetes • Increased competence in hypoglycaemia, hyperglycaemia, insulin use, and glucose management • Reduction in sliding scale insulin use across both facilities • Changes observed in resident HbA1c distribution and medication management
Munshi et al., 2021[20] (USA)	Implementation of a structured diabetes management model of care, with embedded staff education delivered in-person across long-term care facilities.	5	To develop a practical model of care to improve diabetes management in six LTC facilities.	Quantitative Non RCT	6 LTC facilities	• Over 500 staff trained and assessed as competent in diabetes management • Improved identification and management of hypo- and hyperglycaemia • Resolution of hypoglycaemia episodes in 73–90% of cases following staff intervention • Improved diabetes management processes across facilities
Parker et al., 1995[21] (USA)	Face-to-face diabetes education delivered as a series of short lectures over a 12-week period.	4	To create and evaluate a training program for nursing personnel in long-term care facilities.	RCT	4 LTC facilities	• Significant improvement in staff diabetes knowledge in the intervention group • No significant change in observed nursing behaviours related to diabetes care • Educational programme improved theoretical knowledge only
Scott et al., 2011[24] (Scotland)	Printed educational postcards used as informal learning tools during handovers, meetings, and supervision, rather than formal teaching sessions.	5	To improve diabetes knowledge of staff working in care homes.	Mixed Methods	188 Care homes	• Increased staff awareness of diabetes following postcard-based intervention • 27% of homes reported changes in diabetes-related practices • Educational materials used in handovers, meetings, and supervision • No resident-level outcomes assessed
Whylie-Rosett et al., 1985[25] (USA)	Facility-wide diabetes education delivered in person, alongside the introduction of standardised care protocols and charting systems.	3	To improve diabetes care through an educational programme,	Mixed methods	1 large LTC facility (1000 beds)	• Improvements across eight diabetes care measures, including monitoring, documentation, patient knowledge, skills, and compliance • Capillary blood glucose testing replaced urine testing as primary monitoring method • Increased multidisciplinary team involvement in diabetes care • Persistent deficiencies in nutritional documentation and hygiene recording

### Sample

Across all included studies, the primary study participants were care home staff, including registered nurses, nursing aides, healthcare assistants, and unregistered care staff. Sample reporting varied considerably between studies, with some reporting the number of participating care homes and staff, while others also reported resident-level data as part of outcome assessment rather than as direct participants. Where resident numbers were reported, these reflected populations affected by the intervention or outcome measures rather than the recipients of the educational intervention itself. The number of care homes included in the studies ranged from one to 188. A minimum of 2,029 staff participants were reported across studies; however, precise staff numbers were not consistently reported. In several studies, authors reported the number of care homes included without providing detailed information on the corresponding number or characteristics of staff participants, limiting accurate aggregation and comprehensive description of participant demographics.

### Data collection

Various data collection methods were used. Quantitative studies employed pre- and post-tests, knowledge exams, and clinical markers like glycated haemoglobin to assess changes in diabetes care. Parker et al.[21], Lega et al.[19] and Munshi et al.[20] applied these methods. Hare [18] and Gregory [17] emphasised adaptable education and ongoing evaluation, though their data were largely self-reported. Deakin et al.[16] examined long-term knowledge retention through surveys and periodic evaluations.

Mixed-methods studies combined qualitative and quantitative approaches. Hausken et al.[22] used focus group interviews followed by surveys. Heeley-Creed et al.[23] used observations, stakeholder feedback, and evaluations. Scott et al.[24] employed guided discussions, questionnaires, and triangulation for reliability. Whylie-Rosett et al.[25] used interviews, observations, and patient chart audits to document care methods and results.

### Quality appraisal

The quality of papers was assessed using the MMAT scoring system, which rates studies on a scale of five. Study Table 3 highlights the quality appraisal details. A score of five reflects excellence in research design, data collection, analysis, and reporting. Deakin et al.[16], Lega et al.[19], and Munshi et al.[20] had excellent quality scoring 5/5. Hare [18], Hausken et al.[22] and Scott et al.[24] scored 4/5, indicating high quality. Heeley-Creed et al.[23], Parker et al.[21], Whylie-Rosett et al.[25] and Gregory [17] scored 3/5.

### Educational interventions

The ten reviewed studies employed a range of educational interventions, including structured training curricula, that incorporated examinations, training modules, and interactive sessions featuring practical demonstrations. Deakin et al.[16],and Gregory [17] utilised structured modules with examinations and practical demonstrations, though with differing formats and durations. Hare [18] and Hausken et al.[22] conducted needs assessments to create customised material, while Heeley-Creed et al.[23] implemented workshops, conferences, and training sessions to deliver their educational interventions. Munshi et al.[20] and Whylie-Rosett et al.[25] offered longer-term training through Diabetes Care Coordinators and interdisciplinary programmes, respectively, while Parker et al.[21] delivered short lectures over 12 weeks. Scott et al.[24] used seasonal postcards as pedagogical aids which were distributed to the care facilities to stimulate discussions.

### Study results

The results are presented thematically following narrative synthesis. The three themes collectively address the stated review aims. Specifically, Theme 1 summarises the types of educational and training interventions delivered in care homes and explores their impact on diabetes care practice. Theme 2 focuses on the outcomes of these educational interventions in relation to staff knowledge, confidence, and perceived care quality. Theme 3 identifies gaps and limitations within existing educational interventions, including barriers to implementation and areas insufficiently addressed in current training provision.

### Theme 1: impact of diabetes education for practice

This theme addresses the review aims of summarising existing educational and training interventions for diabetes care in care homes and exploring their impact on diabetes care practice. The analysis of the ten research papers included in this review reveals a clear consensus and that is that diabetes education interventions can have a positive impact on and enhance care practices in care homes. These benefits are visible in several areas, including professional development, medication management, patient safety, and interdisciplinary coordination.

Educational interventions are critical to improving professional development among care home staff. Deakin et al.[16] found that staff that received diabetes education had a significant increase in diabetes knowledge over a 12-month period. This steady increase in understanding resulted in continual improvements to diabetic residents’ care regimens, for example greater adherence to glucose monitoring. Similarly, Hausken et al.[22] found that training programmes led to professional updates, a better grasp of diabetes care, and an improved ability to discuss and execute changes in practice. Staff reported an increase in confidence which they attributed to the educational intervention and stated they felt more secure in their knowledge and were better equipped to advocate for their patients. The staff also reported it improved their ability to ask critical questions and make educated judgements, hence improving overall quality of care delivered to residents.

Educational interventions also resulted in significant improvements in medication management in care homes. Lega et al.[19] discovered that after receiving education about diabetes that there was a decrease in the usage of sliding scale insulin and fewer residents requiring renal-based dose changes for glucose-lowering drugs, implying that staff were better able to manage and provide diabetes medications. Furthermore, Munshi et al.[20] found that introducing a realistic diabetes management approach improved targeted glucose monitoring and early interventions, resulting in fewer occurrences of hypoglycaemia and hyperglycaemia. In this study, enhanced staff education significantly improved medication management in care homes. Following the training, insulin adjustments were made in response to blood sugar levels: long-term insulin was adjusted in 55% of low blood sugar (hypoglycaemic) events and short-term insulin in 34% of these events. Similarly, for high blood sugar (hyperglycaemic) events, long-term insulin was adjusted in 60% of cases and short-term insulin in 32% of cases. These adjustments contributed to better patient outcomes, greater glucose control and more effective diabetes treatment overall.

Educational initiatives also had a positive impact on patient safety. Gregory [17] showed a significant reduction in hospital admissions (65%) and emergency calls (69%) relating to diabetic emergencies post-intervention. This was obtained by supporting the delegation of insulin administration to care home staff which resulted in a 378% reduction in weekly visits by district nurses. By delegating insulin administration to trained unregistered practitioners, community nursing visits were reduced, saving approximately £18,900. This change in practice not only optimised resources, but also improved patient safety [17]. Empowering staff with the essential skills and knowledge to administer insulin enabled care homes to provide more responsive and effective diabetes care.

Educational interventions also have the potential to improve interdisciplinary coordination and policy improvements in some care homes. Whylie-Rosett et al.[25] discovered that adding new chart forms and protocols greatly enhanced multidisciplinary coordination, with two-thirds of charts indicating participation from all team members, compared to none prior to intervention. This improved coordination led to fewer hospital admissions and higher overall care quality. Hausken et al.[22] reported regulatory improvements that enabled specialised healthcare personnel to address complex diabetes patients, freeing up care home staff to focus on other residents. These regulatory reforms, prompted by educational interventions, highlight the usefulness of interdisciplinary collaboration in efficiently managing diabetes. In summary, the evaluated research consistently shows that educational interventions increase professional growth, medication management, patient safety, and interdisciplinary coordination. These modifications appear to have the potential to contribute to improved glycaemic management, fewer hospital admissions, and better overall patient care.

### Theme 2: education increases staff knowledge, confidence, and care quality

This theme addresses the review aim of exploring the outcomes of educational interventions for care home staff, with a particular focus on knowledge, confidence, and perceived care quality. Improved knowledge and confidence emerged as a key theme across ten studies, showing the impact of educational interventions on diabetes care in care homes. Nine studies [16–24] reported that these interventions significantly enhanced staff knowledge and confidence, leading to better resident care, as discussed in theme one.

The educational interventions enhanced staff knowledge of various aspects of diabetes care, including distinguishing between type 1 and type 2 diabetes [17, 19–22],

monitoring blood glucose and urine analysis [16, 19]– [20, 23], identifying hypo/hyperglycaemia[19, 20, 22, 24], recognising long-term complications [18–20], and following dietary recommendations for elderly patients [19, 22, 24]. This training enabled staff to make informed decisions and deliver personalised care. In Hausken & Graue [22], confidence scores increased from 7.2 to 8.1 in assessments and from 7.4 to 8.4 in delivering care, with participants reporting fewer errors. Similarly, Parker et al.[21] found a significant increase in post-test scores (73%) compared to pre-test scores (67%) in the treatment group, while the control group showed no change. The educational program effectively increased staff knowledge (F Ratio 8.16, *P*=.006).

Increased staff knowledge led to practical expertise and greater confidence among care home staff. Several studies [16, 19, 20, 23] showed improved confidence in tasks like insulin administration, understanding injection sites, and applying proper procedures. Deakin et al.[16] reported a 20% rise in self-reported confidence and a 15% improvement in understanding injection sites after training. Lega et al.[19] found correct insulin management responses rose from 50% to 91%, while Munshi et al.[20] reported hypoglycaemia cases resolved without recurrence in 73% to 90% of cases due to education. Educational initiatives improved staff’s understanding of diabetes care. Deakin et al.[16] found that 100% of staff valued the diabetes education sessions, while Scott et al.[24] reported 81% found the intervention useful, particularly the postcard idea. Staff felt more prepared to seek help when managing unwell diabetic residents [17, 19].

Increased knowledge and confidence led to improvements in diabetes care. Deakin et al.[16] found staff had a better understanding of management, including medication administration, and became more proficient in blood glucose and urine analysis, resulting in more consistent readings. Munshi et al.[20] similarly reported that instructional interventions improved staff’s understanding of diabetes management and reduced errors in medication administration. Staff also noted improved ability to monitor blood glucose, leading to better management. These enhancements were associated with improved day-to-day diabetes management in care homes, including more consistent monitoring, safer medication practices, and better management of acute glycaemic events, rather than prevention of established long-term diabetes complications. Given that many care home residents already live with established diabetes-related complications, the benefits of improved glycaemic management in this context relate primarily to symptom control and reduction of care-related adverse events, such as hypoglycaemia, infections, and falls.

Educational programs promoted continuous learning and professional development [24]. These interventions improved staff development in care homes. Heeley-Creed et al.[23] reported that 70% of staff involved in the education expressed interest in further sessions and professional development. Parker et al.[21] found 78% of staff gained confidence in appraising literature, 65% were more likely to implement evidence-based practices, and there was a 50% increase in participation in research and development. Overall, the studies suggest educational interventions positively impact staff knowledge and confidence in supporting residents with diabetes.

### Theme 3: Facilitators and barriers to diabetes training in care homes

This theme addresses the review aim of identifying gaps and limitations in current educational interventions for diabetes care in care homes. Seven of the ten studies evaluated [16–18, 20, 22, 24, 25] give useful information about these factors, particularly in the context of diabetes for care home staff. Effective educational initiatives that emphasise coordinated cooperation and well-designed content have been found to considerably improve staff knowledge and care practices. This efficacy appears to be reinforced when employees actively attend these training sessions.

Furthermore, according to Gregory [17] the involvement of multiple stakeholders is critical to the success of these training programs as collaboration among general practitioners (GPs), Clinical Commissioning Groups (CCGs), community nursing teams, practice nurses, chemists and care home employees can result in more extensive and effective training programmes. This study reported post-intervention there was a 40% increase in collaborative meetings between care home staff and other members of the multi-disciplinary team and training sessions saw a 50% rise in attendance when multiple stakeholders were involved. Interactive training methods, such as group discussions, practical demonstrations, visual aids, and games, have also been demonstrated to increase learner engagement and improve overall learning outcomes with Heeley-Creed et al.[23] reporting 80% of participants rated the interactive training methods as more enjoyable and effective compared to traditional methods.

These training programmes face several obstacles. Heeley-Creed et al.[23], Munshi et al.[20] and Deakin et al.[16] found that time and funding limitations often restrict the scope of these initiatives. Deakin et al.[16] noted that inadequate resources confined their study to one facility, while Heeley-Creed et al.[23] and Munshi et al.[20] reported that economic constraints hinder diabetic training in care settings. Staff turnover is another major challenge, as high rates disrupt training continuity, requiring ongoing education, which is hard to maintain without extra time and funding. Geographical challenges, such as rural locations and poor transport links, further complicate training access [23]. In conclusion, while there are ways to improve diabetes training, significant barriers remain.

## Discussion

This scoping review aimed to summarise and present the current literature on educational interventions for optimising diabetes care in care homes, while also identifying gaps in the existing research. This is the first evidence synthesis examining educational interventions for optimising diabetes care in care homes. Diabetes care in the care home setting is inherently complex due to the multifaceted needs of elderly residents, many of whom have multiple comorbidities. Proper management of diabetes in these environments is critical [26–28] as poor glycaemic control can lead to significant complications [29]– [30], including hypoglycaemia [31], cardiovascular issues [32], and impaired wound healing [33]. A major barrier to effective diabetes care identified in this review is the inconsistency in staff training and education [34]. Many care home workers lack the necessary knowledge and skills to properly manage diabetes [35], which undermines their ability to deliver safe and effective care [17, 20]. This deficiency is further compounded by the absence of structured education programmes tailored to the specific needs of care home staff [36], which is critical in ensuring effective care delivery [37].

The findings from this review also highlight the importance of specialised training to address these knowledge gaps [38]. Research has demonstrated that targeted educational interventions can improve the clinical skills of care home staff [39]– [40], particularly in areas such as insulin administration, glycaemic control, and the management of acute complications like hypoglycaemia [41]. For instance, studies have shown that staff education leads to better clinical outcomes for residents, such as improved glycaemic control and reduced hospital admissions due to diabetes-related complications [16, 20, 23].

However, despite the evident benefits of education [42], there are still significant challenges in ensuring consistent and comprehensive staff training [43]. Many care homes face resource constraints that limit their ability to provide ongoing professional development [44]. Additionally, high staff turnover, a common issue in the care home sector [45], can further hinder the implementation of sustainable educational initiatives [46]. As a result, care homes often struggle to maintain a workforce with the necessary skills and knowledge [47] to provide high-quality diabetes care. Addressing these barriers requires a more robust commitment to education, both from individual care homes and at a policy level [48].

The National Advisory Panel for Care Home Diabetes [49] was established in the United Kingdom in 2020 in direct response to the profound impact of COVID-19 on care home residents, particularly those living with diabetes. Recognising that individuals with diabetes are already at an elevated risk due to their frailty and comorbidities, the pandemic exacerbated their vulnerability, resulting in increased morbidity and mortality rates. The establishment of this panel to provide guidance on diabetes education is a positive step, grounded in a consultative approach and informed by input from various stakeholders. The panel highlights essential themes within diabetes education, suggesting a framework for improving training for care home staff. However, a critical gap remains in the existing interventions included in this review: they often lack comprehensive coverage of crucial diabetes-related aspects, such as eye care and foot care. These omissions are significant, as diabetes management in care homes requires holistic attention to such areas to prevent complications and support overall resident health. A notable omission across the included studies was the absence of explicit reporting on foot assessment and foot-protective nursing interventions as outcomes of educational programmes. This is particularly important given the high prevalence of peripheral neuropathy, vascular disease, and diabetes-related foot problems among older adults living in care homes. Failure to address foot care within staff education may limit early identification of foot pathology and the implementation of preventative measures, potentially increasing the risk of avoidable infections, mobility decline, falls, and hospital admission [49]. This gap suggests that existing educational interventions may not fully reflect the holistic and preventative aspects of diabetes care required in care home settings. Future educational programmes should therefore explicitly include foot assessment, risk stratification, and routine foot care practices, and evaluate their impact on staff competence and resident safety. Additionally, this review reveals an absence of digital education methods, which is surprising given the transformative potential digital platforms offer in healthcare training [50–57]. Digital education could provide more accessible, scalable, and flexible training opportunities for care home staff, allowing for interactive, regularly updated, and personalised learning experiences.

The discussion of diabetes management is not confined to the UK alone; other countries, such as Australia, have also made significant strides in enhancing diabetes management in residential aged care facilities. The findings from the narrative review highlight critical themes regarding the impact of diabetes education in care homes, which align closely with the principles outlined in The Diabetes Management in Aged Care: A Practical Handbook [58]. The DMAC emphasises structured diabetes education tailored for aged care staff, demonstrating how such training can significantly enhance staff knowledge, confidence, and ultimately the quality of care provided to residents with diabetes. By offering practical guidance on essential management topics, the DMAC facilitates improved adherence to best practices, which is corroborated by the review’s findings that educated staff are more likely to deliver effective care and achieve better clinical outcomes. Furthermore, the DMAC addresses both facilitators and barriers to implementing diabetes training in residential aged care facilities, identifying strong clinical leadership and staff engagement as key enablers while acknowledging challenges such as high turnover rates and time constraints. This comprehensive approach underscores the necessity for continuous education and support for healthcare providers, reinforcing the notion that effective diabetes management in aged care is contingent upon a well-informed and confident workforce capable of navigating the complexities of resident care. Thus, the DMAC serves as a crucial resource that not only aligns with but also enhances the themes identified in the narrative review, ultimately contributing to improved diabetes outcomes among older adults in residential settings.

The collaborative initiative between the European Geriatric Medicine Society and the European Diabetes Working Party for Older People to develop comprehensive guidelines for managing type 2 diabetes mellitus (T2DM) [59] in community-based adults closely aligns with the themes identified in this review. These guidelines emphasise a holistic approach, prioritising comprehensive assessments and individualised care planning, which supports the review’s findings on the impact of diabetes education on practice. By promoting a multi-disciplinary approach involving diverse healthcare professionals, such as medical practitioners, dieticians, and nurses, the guidelines enhance staff knowledge and confidence, leading to improved care quality for older adults with diabetes. Additionally, they address critical facilitators and barriers by highlighting the importance of screening for frailty and cognitive impairment, ensuring that care encompasses the broader health context of older patients. With approximately 50% of diabetes patients in Europe over 65 years old and rising prevalence rates of T2DM among older adults, these guidelines serve as essential resources for health and social care professionals. They reinforce the need for future interventions to build on these insights and address gaps in training and support for care home staff. Overall, this European initiative exemplifies a concerted effort to elevate diabetes care standards, echoing the narrative review’s emphasis on targeted education and collaborative practice to meet the complex needs of vulnerable populations effectively.

The initiatives in the United States, particularly through the American Diabetes Association (ADA) and its Standards of Care [60], align closely with the themes identified in the narrative review regarding diabetes education and its impact on practice in care homes. The ADA emphasises a person-centred team care model, which enhances the quality of diabetes management among older adults and supports the review’s theme on the impact of diabetes education for practice by promoting collaboration among healthcare providers. This multi-disciplinary approach fosters increased staff knowledge and confidence, thereby improving overall care quality, which aligns with the review’s finding that education enhances staff competence in managing diabetes. Furthermore, the ADA addresses barriers to effective training by advocating for community involvement and support systems that facilitate access to resources. The guidelines also consider social determinants of health, reinforcing the need for comprehensive training that prepares staff to meet the complex needs of residents. Overall, the ADA’s initiatives exemplify a robust framework for improving diabetes care standards in the U.S., reflecting a commitment to evidence-based practices that resonate with the key themes identified in the narrative review. Given the global focus on diabetes management in care homes, future interventions must build on the identified insights to address the identified gaps and ensure that care home staff are fully equipped to meet the complex needs of residents with diabetes.

### Strengths and limitations

This review is, to our knowledge, the first evidence synthesis on educational interventions for diabetes care in care homes, contributing meaningfully to the existing evidence base. The scoping review follows the PRISMA-ScR guidelines, enhancing transparency and reproducibility. Its methodology is well-suited for exploring the broad topic of educational interventions, allowing for a comprehensive assessment of available evidence. The systematic search across multiple databases increases the likelihood of capturing relevant studies, and the inclusion of quality appraisal using the MMAT strengthens the review by assessing the methodological robustness of the included studies. However, the review has some limitations. Focusing solely on care homes may exclude studies from other settings that could be relevant, and the restriction to English-language publications may have missed important non-English studies. Additionally, reliance on published literature introduces potential publication bias, as grey literature may not have been captured. Another concern is the age of many reviewed papers, with no recent studies identified, which raises questions about the relevance of findings to current practice. Finally, the rapidly evolving nature of diabetes care and education suggests that some findings may not reflect the most up-to-date practices, potentially limiting their immediate applicability.

## Conclusion

This review highlights several clear gaps in the current evidence base on diabetes education in care homes. Existing educational interventions are limited in number, often dated, and vary considerably in content and intensity. Key areas of diabetes care, including foot assessment and foot-protective care, eye care, and the use of digital or technology-enabled education, are poorly represented or absent within existing programmes. In addition, few studies evaluate outcomes beyond staff knowledge and confidence, with limited assessment of practice-level or resident-relevant outcomes. Future research should prioritise the development and robust evaluation of structured, up-to-date educational interventions that address these gaps, are tailored to the care home context, and assess their impact on both staff practice and resident safety.

## Data Availability

No datasets were generated or analysed during the current study.
